# Exploration common biomarkers and pathogenesis of primary Sjögren’s syndrome and interstitial lung disease by machine learning and weighted gene co-expression networks

**DOI:** 10.1371/journal.pone.0333070

**Published:** 2025-10-06

**Authors:** Jingqi Dong, Zelin Wang, Yanan Xu, Shufen Liang

**Affiliations:** Department of Laboratory, The Second Hospital of Shanxi Medical University, Taiyuan, Shanxi, China; Kurume University School of Medicine: Kurume Daigaku Igakubu Daigakuin Igaku Kenkyuka, JAPAN

## Abstract

**Background:**

Primary Sjögren’s syndrome (pSS) is an autoimmune and inflammatory disorder that may affect the lungs, leading to interstitial lung disease (ILD). However, the diagnosis of progression from pSS to ILD is frequently delayed due to unstandardized interdisciplinary diagnostic criteria and a lack of reliable shared biomarkers. This diagnostic challenge, compounded by significant pathophysiological divergence in target organs, has hindered elucidation of their comorbidity mechanisms. This study employs integrated bioinformatics to identify shared biomarkers in pSS and ILD, deciphers their pathogenic mechanisms, and predicts targeted therapeutics via network pharmacology

**Methods:**

From the Gene Expression Omnibus (GEO) database, we retrieved gene expression profiles of pSS and ILD. Differential expression gene (DEG) analysis was performed on the profiles, followed by further screening using four machine learning algorithms. Concurrently, weighted gene co-expression network analysis (WGCNA) was applied to identify gene modules, and enrichment analysis of WGCNA-derived genes was conducted to explore their biological functions. Genes obtained from WGCNA and machine learning approaches were then intersected to identify candidate biomarkers for pSS-ILD. The diagnostic potential of these candidate genes was evaluated in both discovery and validation sets using receiver operating characteristic (ROC) curves. Finally, we performed immune cell infiltration analysis of candidate genes, regulatory network construction for transcription factor (TF)-gene and miRNA-gene interactions, drug-target prediction, and molecular docking coupled with molecular dynamics simulations for predicted drugs.

**Results:**

Differential expression analysis identified 25 shared genes between pSS and ILD gene expression profiles, with machine learning algorithms refining six key genes from these DEGs. WGCNA revealed 39 intersecting genes significantly enriched in biological processes including cell division, oocyte maturation, and metabolic regulation. Intersection of machine learning and WGCNA results yielded two hub genes (CYSLTR1 and SIGLEC10), both demonstrating robust diagnostic value in discovery and validation cohorts. Immune cell infiltration profiling showed: upregulation of activated CD4+ memory T cells and memory B cells; downregulation of resting NK cells. Regulatory network analysis indicated FOXC1, hsa-mir-27a-3p, hsa-mir-195-5p, and hsa-miR-26a-5p as potential coregulators of CYSLTR1 and SIGLEC10 expression. Finally, ten candidate drug compounds targeting the hub genes were prioritized, exemplified by:Rev-5901 (CTD 00002161), Zafirlukast (BOSS database) and Montelukast (CTD 00003205). Molecular docking demonstrated substantial binding affinity of both montelukast and zafirlukast for CYSLTR1, while molecular dynamics simulations further validated the stability of their complexes.

**Conclusion:**

This study revealed that CYSLTR1 and SIGLEC10 demonstrate diagnostic potential for pSS-ILD. Their mechanism of action likely involves synergistically upregulating memory B cells to promote disease progression. Furthermore, we identified montelukast as a potential therapeutic agent. This discovery holds promise for improving clinical outcomes for pSS-ILD patients.

## 1. Introduction

Primary Sjögren’s syndrome (pSS) is a systemic autoimmune disorder characterized by lymphocyte infiltration of exocrine glands [1], particularly the salivary and lacrimal glands. This immune-mediated process leads to progressive glandular dysfunction, culminating in xerostomia and keratoconjunctivitis sicca [2]. Extraglandular manifestations include arthralgia, fatigue, synovitis, neuropathy, and respiratory symptoms such as dyspnea and cough [3]. pSS arises from the interplay of genetic, viral, and inflammatory factors [4], with its heterogeneous clinical spectrum causing significant long-term deterioration in patients’ quality of life. The underlying pathomechanisms remain incompletely understood.

Interstitial lung disease (ILD) refers to a group of diffuse pulmonary disorders affecting alveoli, pulmonary interstitium, and bronchioles. Pathologically defined by varying degrees of interstitial inflammation and fibrosis, ILD ultimately causes parenchymal damage [5]. Its classification system is heterogeneous, encompassing connective tissue disease-associated ILD (CTD-ILD), idiopathic interstitial pneumonia (IIP), exposure-related ILD, and other rare subtypes [6]. While recent years have witnessed growing focus on the pathogenesis of CTD-ILD [7–9], research on pSS-associated ILD (pSS-ILD) predominantly relies on clinical evaluations and systematic analyses based on imaging and histopathology, leaving its core pathogenic mechanisms elusive.

Respiratory involvement represents the most frequent extraglandular manifestation in pSS, with the incidence of interstitial lung disease (ILD) ranging from 6% to 23% [10,11]. Although pSS-ILD is often considered indolent, a retrospective study by Gao et al. demonstrated that 25% of patients died during follow-up, with respiratory failure accounting for 61% of mortality [12], establishing ILD as a severe and life-threatening complication of pSS. ILD often described as a late complication of pSS, the prevalence of ILD increases with disease duration [7]. This phenomenon may reflect diagnostic sensitivity improvements, as technological advances in pulmonary assessment are narrowing the age gap between pSS and ILD diagnoses [13]. Consequently, early detection of pSS-ILD poses a major clinical challenge and is critical for therapeutic management and prognosis. Current diagnosis relies primarily on high-resolution computed tomography (HRCT), abnormal pulmonary function tests (PFTs), and histopathological evidence from lung biopsies [14]. HRCT exhibits superior sensitivity over PFTs, increasing ILD detection rates from 16% to 25% [10]. However, due to factors such as radiation exposure from imaging examinations and poor patient compliance with invasive procedures, the application of ILD for early diagnosis in the course of pSS is limited. Various biomarkers, serving as a non-invasive screening method, are emerging as promising tools for screening pSS-ILD. Recently, KL-6 has been identified as a serum biomarker associated with both detection and severity assessment of CTD-ILD [15]. Additionally, multiple studies recognize anti-Ro52 antibody as an independent risk factor for pSS-ILD progression [16–18]. Recent years have seen a proliferation of therapeutic interventions for pSS-ILD, predominantly palliative approaches. However, the complex multifactorial pathogenesis of pSS-ILD compromises drug target specificity, necessitating further mechanistic investigation. For instance, while combining antifibrotic agents with conventional immunosuppressants represents the current therapeutic paradigm, its non-selective nature yields suboptimal efficacy in specific ILD subtypes [19].

IIP encompass subtypes such as idiopathic pulmonary fibrosis (IPF) and nonspecific interstitial pneumonia (NSIP). Recent studies have revealed associations in pathogenesis and overlapping pathological features between IPF and other ILDs. Renzoni et al. found consistent MUC5B staining in the airways and honeycombing areas of IPF and other ILDs, with similar radiological and histological findings [20]; Hoffmann-Vold et al. demonstrated that during the progression of both IPF and CTD-ILD, PDGF-AA, PDGF-BB, M-CSF, and VEGF exhibit consistent trends of change, collectively driving pathological alterations. These alterations include growth factor activation, changes in cytokine and chemokine levels, vascular remodeling, and epigenetic reprogramming of fibroblasts, among others [21]; Furthermore, Distler and his team discovered that IPF and other ILDs share common activation responses in pro-fibrotic signaling pathways, including platelet-derived growth factor (PDGF), transforming growth factor-β (TGFβ), hedgehog signalling, and WNT signalling [22]. Consequently, despite differing initial etiologies, IPF and other ILDs may progress through shared molecular pathways. NSIP is the most common pattern in primary pSS-ILD [23]. It can be secondary to connective tissue disease (CTD-NSIP) or idiopathic (INSIP) [24]. CTD-NSIP and INSIP share some similarities in clinical manifestations and imaging features. Notably, ILD may be the initial presenting symptom of CTD, and follow-up studies have found that ultimately 15% of patients with INSIP are diagnosed with CTD-NSIP [25]. Given the overlapping pathogenic mechanisms between IIP and other ILD, we aim to explore potential shared molecular pathways between IIP and pSS, thereby addressing the knowledge gap in the field of pSS-ILD.

Critical gaps persist in elucidating shared molecular mechanisms between pSS and ILD, impeding targeted therapeutic development. For instance, while pSS salivary glands feature B-cell infiltration and germinal center formation, ILD lung tissues exhibit Th1/Th17 polarization and macrophage recruitment, yet the mechanisms governing immune cell migration and phenotypic conversion between these sites remain unelucidated [26]. Key molecular switches, such as the drivers of inflammation-to-fibrosis transition, remain unidentified, and proposed serum biomarkers for pSS-ILD lack integration with mechanistic pathways and computational validation [27]. These deficits directly contribute to delayed diagnosis and non-targeted therapeutic approaches.

**Fig 1:**
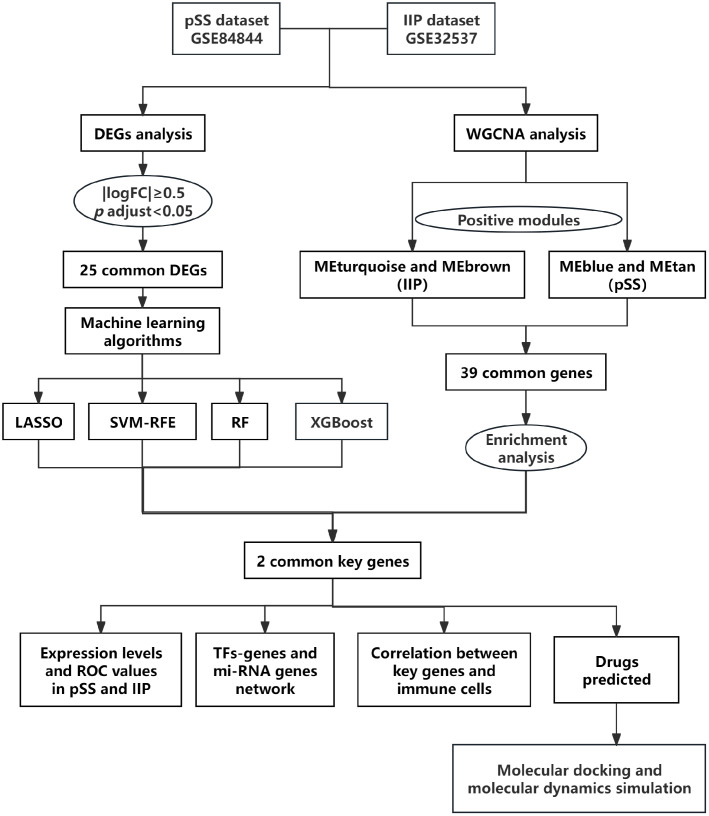
Study flowchart.

To establish an early diagnostic basis and investigate pathogenic pathways leveraging biomarkers for pSS-ILD, this study integrated gene expression profiles of pSS and IIP from Gene Expression Omnibus (GEO) databases, then employed differential expression analysis, machine learning, and weighted gene co-expression network analysis (WGCNA) to identify hub genes. Subsequent analyses included functional enrichment, immune cell infiltration assessment, TF-gene regulatory networks, and miRNA-gene interactions. Potential therapeutic agents were screened through computational approaches The study workflow is illustrated in Fig 1. Based on this bioinformatic analysis, we aimed to screen key pathogenic genes to elucidate the pathogenesis of pSS-ILD. This approach not only provides a framework for future research but also identifies promising therapeutic drug candidates, ultimately aiming to improve patients’ quality of life.

## 2. Materials and methods

### 2.1. Data sources

The Gene Expression Omnibus (GEO) database (https://www.ncbi.nlm.nih.gov/geo/) was used to acquire gene expression datasets for pSS and IIP [28]. According to the search criteria as follows: (1) *Homo sapiens* was the source of the samples, (2) the dataset encompassed both case and control groups, (3) a minimum of 10 samples per group. Four gene expression datasets were finally obtained, namely GSE84844, GSE66795, GSE32537 and GSE110147 (Table 1). Among them, the dataset of GSE84844 consisted of 30 pSS samples and 30 healthy samples.The dataset of GSE66795 consisted of 131 pSS samples and 29 healthy samples.The dataset of GSE32537 consisted of 167 IIP samples and 50 healthy samples.The dataset of GSE110147 consisted of 37 IIP samples and 11 healthy samples. For our analysis, GSE84844 and GSE32537 were designated as the discovery sets, while GSE66795 and GSE110147 served as the validation sets.

**Table 1:** An overview of the GEO datasets utilized in this study.

GSE number	Platform	Cases	Controls	Tissue	Disease	Group
GSE84844	GPL570	30	30	Peripheral blood	pSS	Discovery
GSE66795	GPL10558	131	29	Peripheral blood	pSS	Validation
GSE32537	GPL6244	167	50	Lung tissue	IIP	Discovery
GSE110147	GPL6244	37	11	Lung tissue	IIP	Validation

### 2.2. Identification of differentially expressed genes (DEGs)

GSE84844 and GSE32537 were standardized using the “limma” and “pheatmap” packages of R software (version 4.4.1). Differences were analyzed between the case and healthy groups, and to identify DEGs, the screening criteria were set as adjusted *p*-values < 0.05 and |log2-fold change (FC)| ≥ 0.5. The Volcanograms of DEGs were plotted utilizing the “ggvolcano” package. The common DEGs of pSS and IIP were obtained using the Venn diagram.

### 2.3. Machine learning screening of hub genes

This study employed four machine learning algorithms for feature selection: Support Vector Machines-Recursive Feature Elimination (SVM-RFE), Least Absolute Shrinkage and Selection Operator (LASSO), Random Forest (RF), and XGBoost. SVM-RFE algorithm is implemented via the “e1071” package, screening features based on their correlation with target classes. LASSO regression is executed using the “glmnet” package in R, which applies L1 regularization to shrink coefficients of non-critical variables to zero for feature selection. RF is analyzed using the “randomForest” package, which ranks features by calculating importance scores. XGBoost algorithm is applied through the “xgboost” package, involving multiple iterations to compute global feature importance scores by weighting the frequency and quality of feature splits in decision trees.

The intersection of genes identified by all four algorithms was visualized using a Venn diagram, and these overlapping genes were designated as core genes for the pSS and IIP datasets.

### 2.4. Weighted gene co-expression network analysis (WGCNA) and enrichment analysis

Using the “WGCNA” package in R software to identify highly correlated co-expressed gene modules [29]. First, for subsequent analysis, we selected genes that exhibited variance scores within the top 25%. Second, the data were processed to remove the outlier samples. Third, utilizing the “select soft threshold” feature, we constructed a scale-free network to identify the optimal soft-threshold power β. Fourth, a topological overlap matrix (TOM) was generated based on the neighbor-joining matrix. Fifth, co-expressed gene modules were classified using average hierarchical clustering and dynamic tree-cutting methods. Sixth, to evaluate the relationship between modules and clinical features, we calculated gene significance (GS) and module membership (MM), and the most strongly correlated and positively correlated modules were extracted for further gene information analysis. Finally, pSS gene modules were intersected with IIP gene modules using a Venn diagram to obtain the intersected genes.

A key advantage of WGCNA lies in its ability to directly associate these co-expression modules with clinically relevant traits. These modules naturally represent biologically meaningful gene sets with evidence of coordinated expression. Enrichment analysis applied to these modules directly interrogates the collective biological function of the putative co-regulated gene sets. Therefore, we utilized the “clusterProfiler” and “ggplot2” packages to perform functional enrichment analysis and visualization on the genes filtered by WGCNA. Functional enrichment analysis encompasses both Gene Ontology (GO) and Kyoto Encyclopedia of Genes and Genomes (KEGG) analyses. The GO analysis is divided into three main categories: biological process (BP), cellular component (CC), and molecular function (MF), while The KEGG analysis provides a comprehensive investigation into gene functions, highlighting statistically significant pathways that are enriched in genes.

### 2.5. Identification of diagnostic biomarkers as well as expression analysis and diagnostic evaluation

In order to learn more about the pathogenesis of pSS-ILD, the hub genes derived from the machine analysis and the genes screened by WGCNA were taken to be intersected, and the resulting genes were considered to be the diagnostic biomarkers of pSS and IIP. We carried out statistical analysis utilizing GraphPad Prism (version 8.0.2) to compare the differences in expression of the diagnostic biomarkers across the four datasets. A column-line graph model was then constructed and calibration curves were plotted using the “rms” R package. Each gene was given a relative expression point, with the “Total Points” indicates the cumulative total of these points for the specified genes. Finally, the “pROC” package was utilized to carry out the receiver operator characteristic (ROC) analysis for diagnostic biomarkers, and to determine the performance of the diagnostic biomarkers in differentiating between diseased and healthy groups, ROC curves were plotted, and the area under the curve (AUC) was used as the evaluation criterion. An adjusted *p*-value < 0.05 was set as the criterion for statistically meaningful.

### 2.6. Analysis of immune cell infiltration

In this study, we used the online website CIBERSORTx (https://cibersortx.stanford.edu/) to obtain the percentage of 22 immune cells in GSE84844 and GSE32537, and the immune cell ratios were visualized utilizing the “ggplot2”, “corrplot”, and “vioplot” packages within the R programming environment. The expression differences of 22 types of immune cells between the two diseases, as well as the correlation analysis of immune cells, were obtained. Subsequently, Spearman correlation analysis was employed to determine the relationship between diagnostic biomarkers and immune cells, with a *p*-value < 0.05 was set as the criterion for statistically significance.

### 2.7. Construction of transcription factors(TFs)-genes and miRNA-genes regulatory networks

TFs and miRNAs for diagnostic biomarkers were searched using the JASPAR database and TarBase database of the NetworkAnalyst 3.0 tool (https://www.networkanalyst.ca/), and network-based visualization of the results was achieved by implementing Cytoscape (version 3.10.0) for regulatory network modeling.

### 2.8. Predicting relevant drugs

Using the “DsigDB” database of the Enrichr platform (https://maayanlab.cloud/Enrichr/enrich), the drugs associated with the predicted diagnostic biomarkers were predicted, and those with *p*-values <0.05 were retained and visualized in a three-line table based on the analytical outcomes.

### 2.9. Molecular docking and molecular dynamics simulation

Protein sequences corresponding to target genes were obtained from the UniProt database. Corresponding crystal structures were subsequently retrieved from the PDB database and preprocessed using PyMOL software. Concurrently, 3D structures of drug compounds were downloaded from PubChem, with OpenBabel employed for format conversion and structural optimization. Molecular docking was performed using AutoDock Vina. Docking results were visualized and analyzed in PyMOL.

Molecular dynamics simulations were conducted using GROMACS 2022.3. Proteins were modeled with the Amber99SB-ILDN force field, small-molecule ligands were parameterized with GAFF2, and solvation was implemented using the TIP3P water model. System preparation involved adding solvent molecules and counterions for charge neutralization. Energy minimization was performed via the steepest descent algorithm, followed by 1,000,000-step equilibration to ensure proper system relaxation. Production simulations were then run for 100 ns under isothermal-isobaric conditions of 300 K and 100 kPa. Based on the trajectories, analyses were performed for root mean square deviation (RMSD), root mean square fluctuation (RMSF), solvent accessible surface area (SASA), radius of gyration (Rg), hydrogen bond occupancy, and free energy changes.

## 3. Results

### 3.1. Identification of DEGs

**Fig 2:**
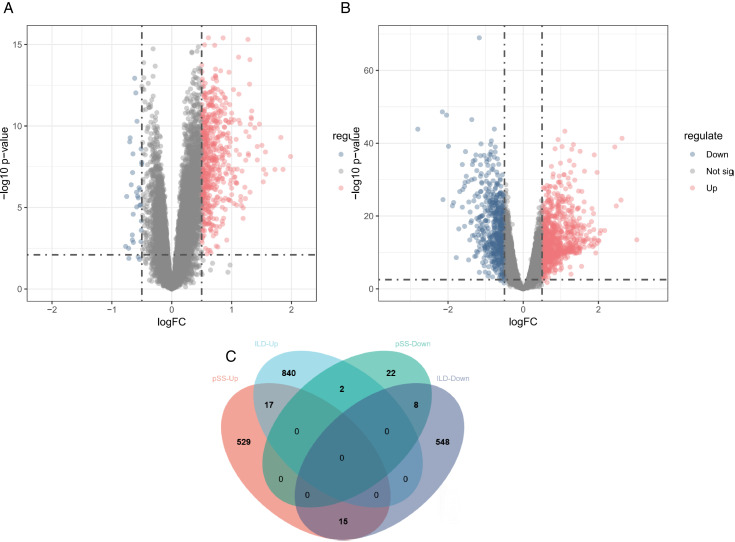
Shared DEGs in pSS (GSE84844) and IIP (GSE32537). (A, B) Volcanograms of DEGs in pSS and IIP. (C) The Venn diagram analysis revealed common DEGs between pSS and IIP. DEGs, differentially expressed genes.

Differential expression analysis of the pSS cohort (GSE84844) uncovered 593 candidate genes (DEGs) exhibiting significant transcriptional alterations, including 561 being over-expressed and 32 being under-expressed (Fig 2A). In the IIP dataset (GSE32537), 1,430 DEGs were identified, with 859 over-expressed and 571 under-expressed genes. (Fig 2B). Using the Venn diagram (Fig 2C), 25 common DEGs were obtained between pSS and IIP, of which 17 were up-expressed and 8 down-expressed.

### 3.2. Hub genes identified through machine learning

**Fig 3:**
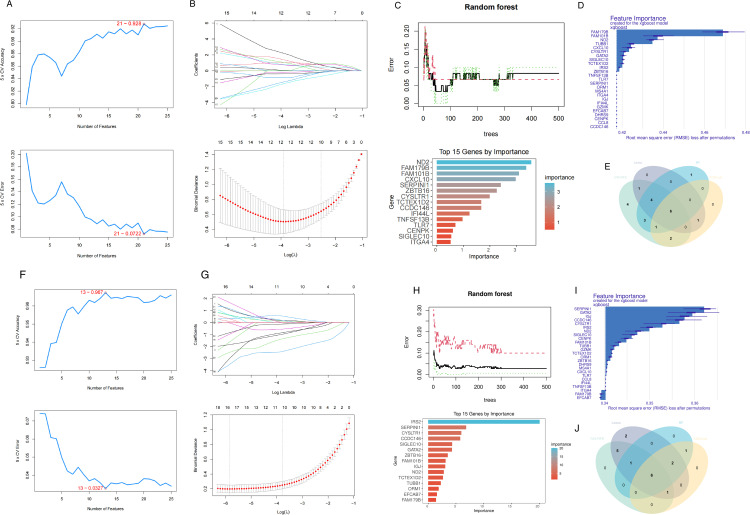
Machine learning in screening hub genes. (A,B,C,D) The results of SVM-RFE algorithm, LASSO regression, RF classifier and XGBoost algorithm for pSS. (E) Venn diagram presents the six genes identified through four distinct algorithms within pSS. (F,G,H,I) The results of SVM-RFE algorithm, LASSO regression, RF classifier and XGBoost algorithm for IIP. (J) Venn diagram presents the six genes identified through four distinct algorithms within IIP.

In the pSS dataset (GSE84844), we selected 21 genes by means of the SVM-RFE algorithm, where the screening conditions were the highest accuracy and the minimum error (Fig 3A). 12 genes were screened out in the LASSO regression algorithm corresponding to the minimum binomial deviation (Fig 3B). In the RF classifier, we ultimately chose the 15 genes that were ranked highest based on their importance. (Fig 3C). XGBoost selected the top 10 genes by importance score (Fig 3D). Finally, the four machine algorithms of the pSS dataset were intersected to obtain 6 overlapping genes (Fig 3E). In the IIP dataset (GSE32537), 13 genes were found by the SVM-RFE algorithm (Fig 3F), 17 genes were screened by LASSO regression (Fig 3G), and the top 15 genes that were ranked highest based on their importance were found by the RF classifier (Fig 3H). XGBoost selected the top 10 importance-ranked genes (Fig 3I). Similarly, 6 overlapping genes were obtained from the IIP dataset (Fig 3J).

### 3.3. Weighted gene co-expression network analysis and enrichment analysis

**Fig 4:**
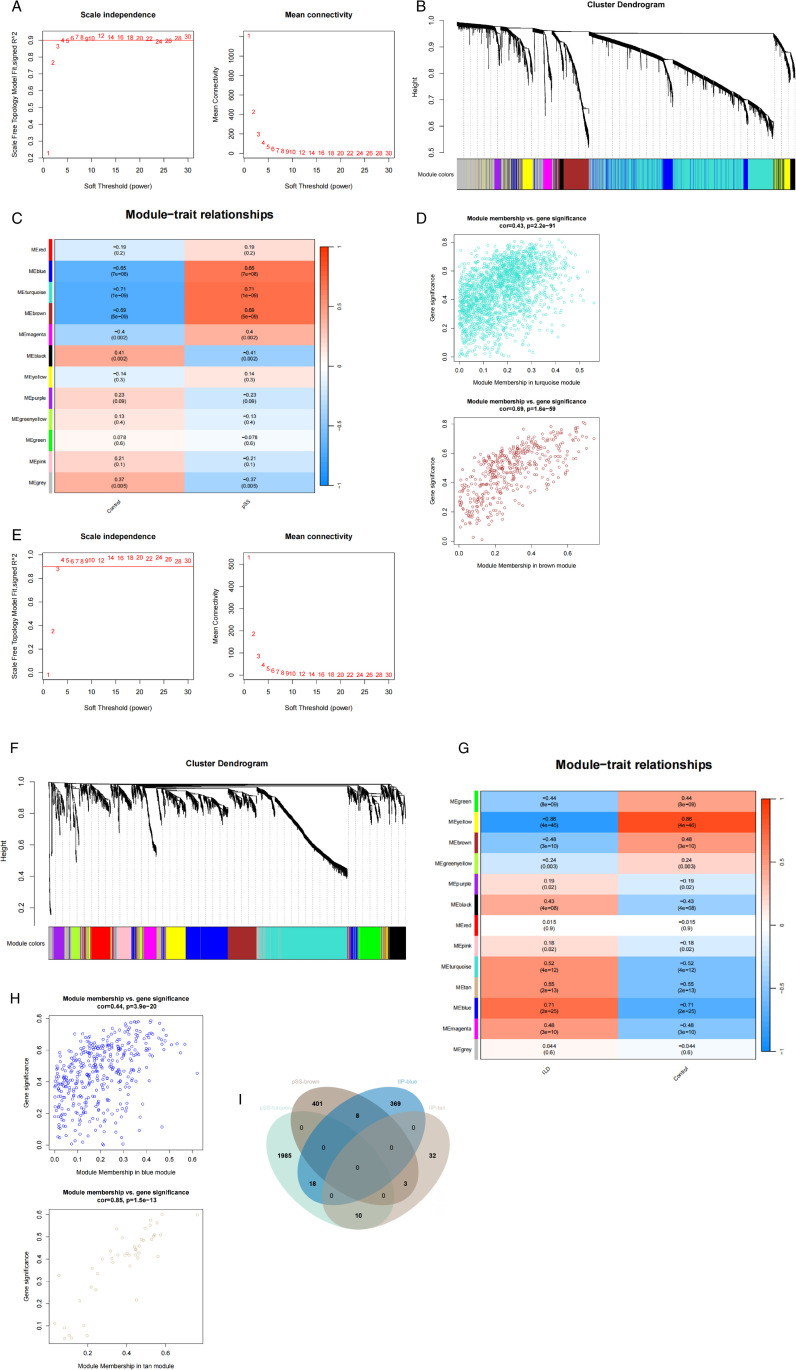
Common gene signatures in pSS dataset (GSE84844) and IIP dataset (GSE32537) datasets using WGCNA algorithm. (A) Determination of optimal soft-thresholding power for pSS analysis. (B) Dendrogram of co-expressed gene clusters identified in pSS datasets. (C) Module-trait association heatmap generated for pSS cohorts. (D) Scatter plot visualization of gene significance-module membership correlations within turquoise and brown co-expression modules in pSS cohorts. (E) Determination of optimal soft-thresholding power for IIP analysis. (F) Dendrogram of co-expressed gene clusters identified in IIP datasets. (G) Module-trait association heatmap generated for IIP cohorts. (H) Scatter plot visualization of gene significance-module membership correlations within blue and tan co-expression modules in IIP cohorts. (I) Comparative Venn analysis of shared genes at the intersection of the two modules of pSS and IIP.

Gene modules exhibiting the highest correlation in the pSS or IIP datasets were screened by the WGCNA method. The pSS dataset (GSE84844) was analyzed with the optimal soft thresholding power was 4. (Fig 4A). The analysis revealed a total of 12 modules, of which the turquoise module (correlation coefficient = 0.71, *p* = 1e-09) and brown module (correlation coefficient = 0.69, *p* = 5e-09) possess 2013 and 412 genes respectively, and exhibit the highest degree of positive correlation with the disease (Figs 4B, C). Furthermore, scatterplots demonstrated a positive correlation between MM and GS for both the turquoise and brown modules (Fig 4D).

Similarly, in the IIP dataset (GSE32537), the optimal soft thresholding power was 4 (Fig 4E). A comprehensive analysis determined a total of 13 modules (Fig 4F, G), and to ensure consistency with the pSS dataset, the blue module (correlation coefficient = 0.71, *p* = 2e-25) and the tan module (correlation coefficient = 0.55, *p* = 2e-13) containing 395 and 45 genes respectively, and exhibit the highest degree of positive correlation with IIP. The module memberships in the blue module and the tan module with gene significance showed positive correlation through scatter plot (Fig 4H). Finally, 39 overlapping genes of the pSS and IIP associated modules were obtained using the Venn diagram (Fig 4I).

**Fig 5:**
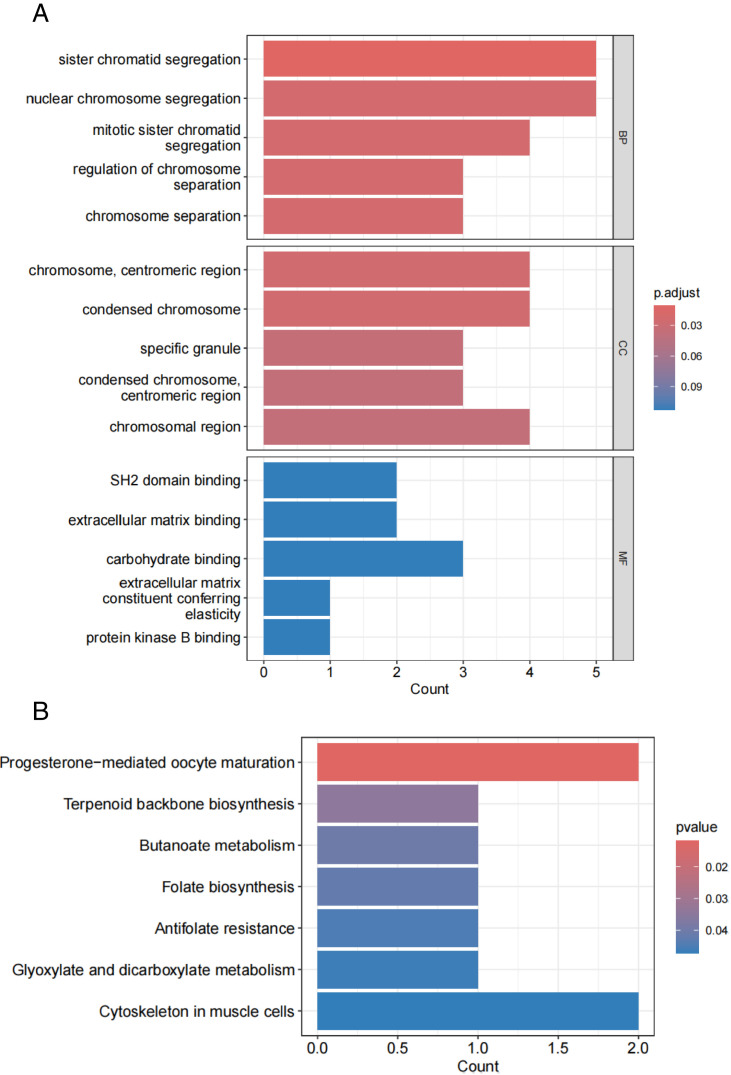
GO and KEGG pathway enrichment analyses of the overlapping genes by WGCNA algorithm. (A) GO enrichment analysis was conducted for the intersecting genes. (B) KEGG enrichment analysis was carried out on the intersecting genes.

To investigate the biological characteristics and pathways of the 39 key genes identified through WGCNA, we conducted Gene Ontology (GO) and Kyoto Encyclopedia of Genes and Genomes (KEGG) pathway enrichment analyses. In each category of the GO analysis, the top five terms are listed (Fig 5A). BP was mainly enriched for sister chromatid segregation, nuclear chromosome segregation, and chromosome separation. CC was mainly enriched for chromosomes, specific granules, condensed chromosomes in the mitotic region, and condensed chromosomes. MF was mainly enriched for extracellular matrix binding, components of the extracellular matrix that confer elasticity, and protein kinase B binding. KEGG pathway enrichment analysis highlighted six pathways primarily associated with progesterone-mediated oocyte maturation, terpene skeleton biosynthesis, and butyric acid metabolism (Fig 5B). These results suggest that pSS and IIP are associated with the processes of cell division, oocyte maturation and metabolic regulation.

### 3.4. Identification of diagnostic biomarkers as well as expression analysis and diagnostic evaluation

**Fig 6:**
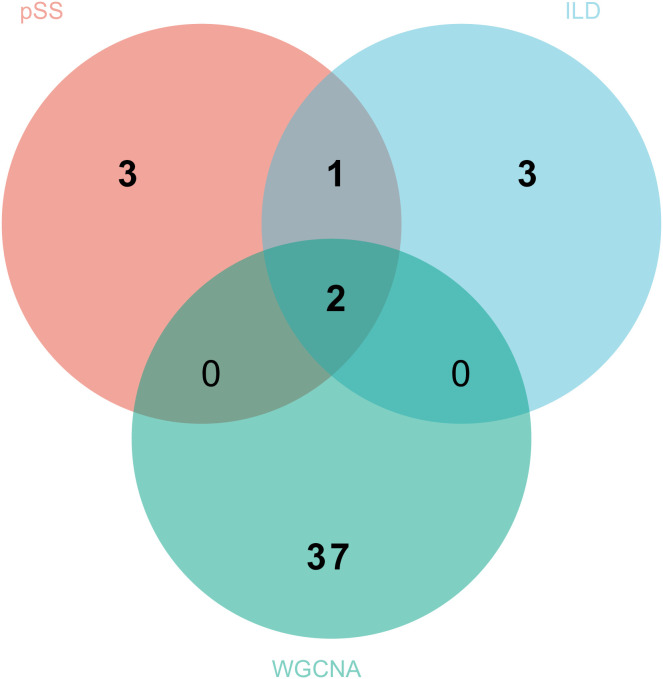
Identification of common key genes by integrating machine learning with WGCNA analysis.

In order to obtain plausible diagnostic biomarkers, two genes, *CYSLTR1* and *SIGLEC10*, obtained as potential diagnostic biomarkers, were taken as intersections of the hub genes obtained by machine learning with the genes screened by WGCNA using a Venn diagram (Fig 6).

**Fig 7:**
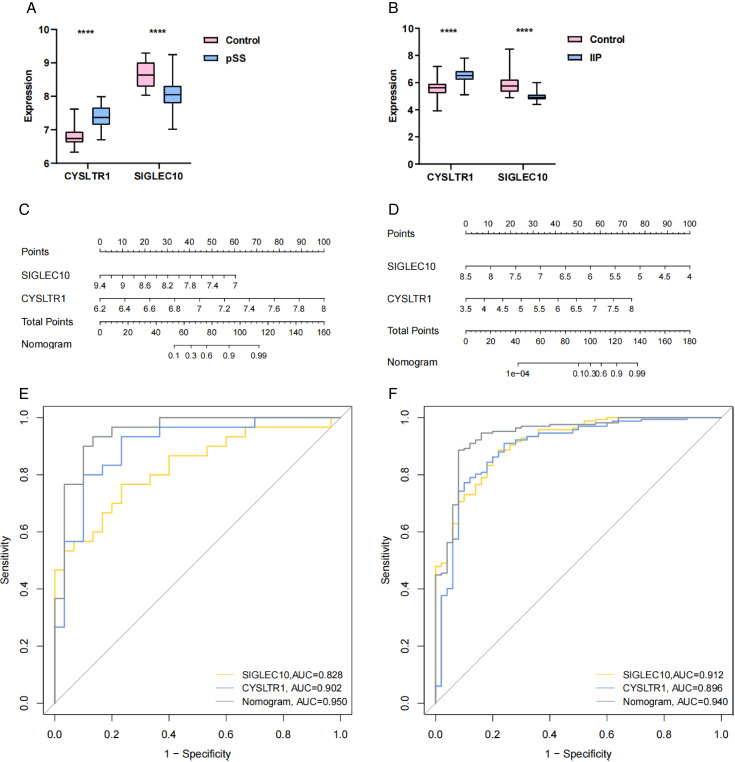
Validation of the key genes in discovery sets. (A, B) The expression levels of the two key genes in the pSS (GSE84844) and the IIP (GSE32537) discovery sets. (C, D) Nomogram construction of the two key genes in the pSS (GSE84844) and the IIP (GSE32537) discovery sets. (E, F) The ROC curves of the two key genes in the pSS (GSE84844) and the IIP (GSE32537) discovery sets. (*p < 0.05; **p < 0.01; ***p < 0.001; ****p < 0.0001).

The accuracy of the above 2 genes as diagnostic biomarkers for pSS and IIP was evaluated by examining their expression levels and diagnostic effects. In the discovery set (GSE84844, GSE32537), the expression level of *SIGLEC10* was significantly lower in the patient cohort than in the control cohort, and the expression of *CYSLTR1* was significantly higher in the disease cohort than in the control cohort (Figs 7A, B). Nomograms of the 2 genes were then plotted, the relative expression scores of each gene were calculated, and the total score was determined (Figs 7C, D). According to the ROC curves, the AUC values of *SIGLEC10* = 0.828 and *CYSLTR1* = 0.902 in the pSS dataset, and *SIGLEC10* = 0.912 and *CYSLTR1* = 0.896 in the IIP dataset, which had good diagnostic performance (Fig 7E, F).

**Fig 8:**
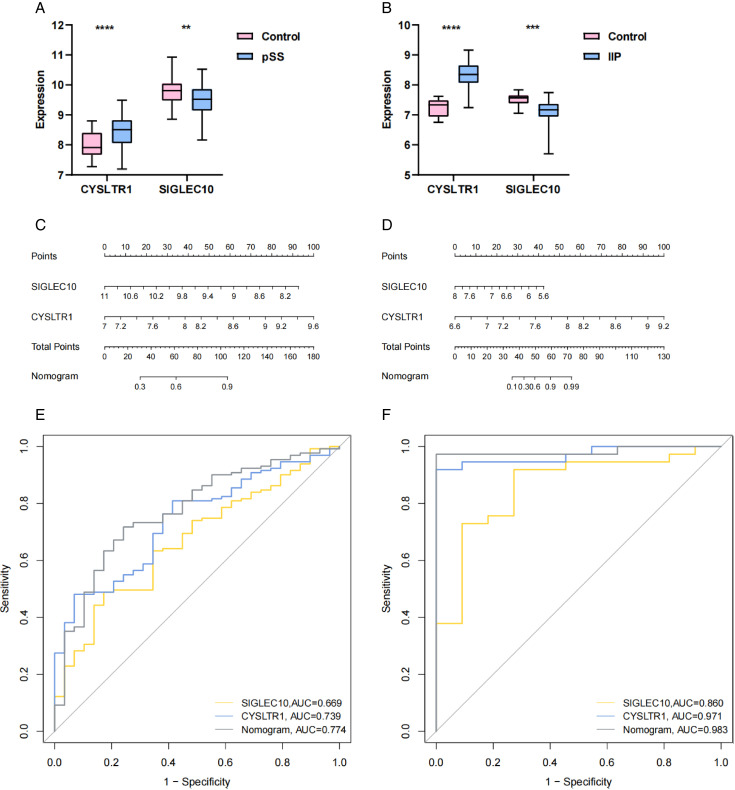
Validation of the key genes in validation cohorts. (A, B) The expression levels of the two key genes in validation cohorts for pSS (GSE66795) and IIP (GSE110147). (C, D) Nomogram construction of the two key genes in validation cohorts for pSS (GSE66795) and IIP (GSE110147). (E, F) The ROC curves of the two key genes in discovery cohorts for pSS (GSE66795) and IIP (GSE110147). (*p < 0.05; **p < 0.01; ***p < 0.001; ****p < 0.0001).

To further examine the capacity of potential diagnostic biomarkers to discriminate between diseased and healthy populations, the validation cohort (GSE66795, GSE110147) was used to assess the differential expression and diagnostic efficacy of the two aforementioned genes. The resultant plots showed that both *CYSLTR1* and *SIGLEC10* had significant expression differences in the validation datasets, and the trend was consistent with the discovery datasets (Fig 8A, B). Similarly, Nomogram prediction models (Fig 8C, D) and ROC curves (Fig 8E, F) were plotted for both genes, showing that both genes show promise as diagnostic biomarkers for pSS and IIP.

### 3.5. Immune cell infiltration analysis

**Fig 9:**
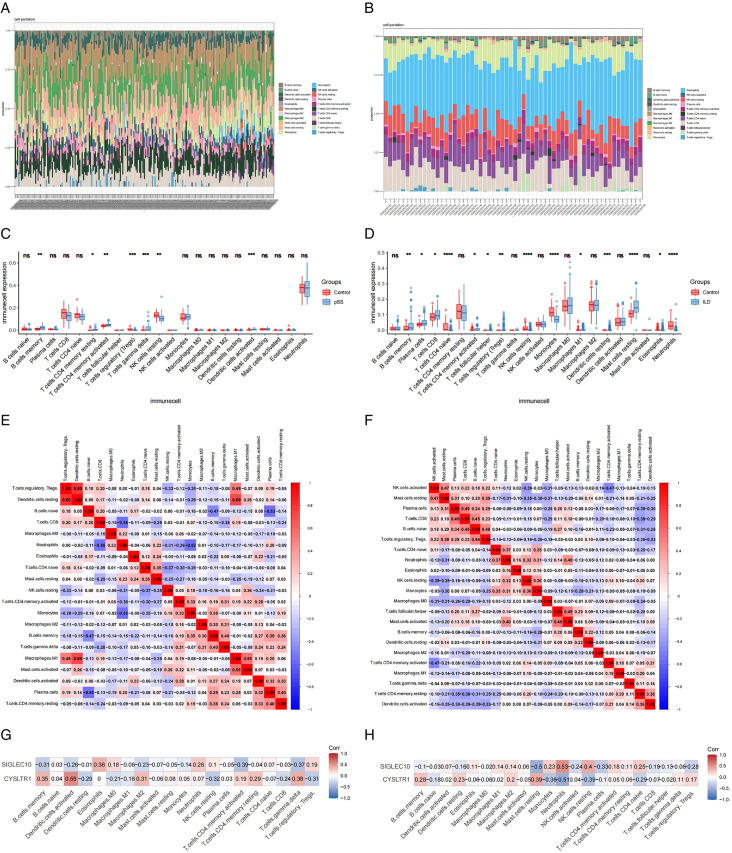
Assessment of immune cell infiltration characteristics. (A, B) The composition of immune cell infiltration in pSS (GSE84844) and IIP(GSE32537). (C, D)Boxplots showing the differences in immune cell infiltration between groups in pSS (GSE84844) and IIP (GSE32537) (*p < 0.05; **p < 0.01; ***p < 0.001; ****p < 0.0001). (E, F) These heatmaps illustrate the relationships among various immune cells in pSS ( GSE84844) and IIP (GSE32537). (G, H) Association between infiltrating immune cells and two diagnostic biomarkers.

We assessed the degree of immune cell infiltration in discovery datasets utilizing the CIBERSORT algorithm, to investigate the association between immune cells and pathological mechanisms in pSS and IIP. The proportions of 22 immune cell types in these datasets were visualized as bar graphs (Figs 9A, B). Box plots illustrating variations in immune cell infiltration revealed that, compared to healthy controls, the pSS group exhibited elevated levels of memory B cells, resting memory CD4 + T cells, activated memory CD4 + T cells, gamma delta T cells, M2 macrophages, and activated dendritic cells, while regulatory T cells (Tregs) and resting NK cells were reduced (Fig 9C). In the IIP group, increased infiltration was observed for memory B cells, plasma cells, CD8 + T cells, activated memory CD4 + T cells, follicular helper T cells, Tregs, resting dendritic cells, and resting mast cells. Conversely, naive CD4 + T cells, resting NK cells, monocytes, M1 macrophages, eosinophils, and neutrophils showed decreased infiltration (Fig 9D). Notably, memory B cells, activated memory CD4 + T cells, and resting NK cells displayed consistent trends in both the pSS and IIP groups.

Furthermore, correlation heatmaps for the 22 immune cell types revealed that in the pSS dataset, resting dendritic cells exhibited positive association with Tregs and M1 macrophages (r = 0.80 and r = 0.69, respectively), while M1 macrophages demonstrated a significant positive association with activated mast cells (r = 0.55). Neutrophils, on the other hand, were inversely associated with CD8 + T cells and monocytes (r = −0.55 and r = −0.52, respectively), and naive B cells were inversely associated with plasma cells (r = −0.53) (Fig 9E). In the IIP dataset, activated NK cells demonstrated a positive correlation with resting mast cells (r = 0.47), plasma cells were positively correlated with CD8 + T cells (r = 0.49), and resting B cells demonstrated a positive correlation with Tregs (r = 0.46). Conversely, activated NK cells were inversely associated with activated memory CD4 + T cells (r = −0.47) (Fig 9F).

Finally, an interaction assessment was carried out to explore associations linking the 2 diagnostic biomarkers and 22 immune cell types. The results revealed that in the pSS dataset (Fig 9G), *CYSLTR1* exhibited positive correlations with memory B cells, activated dendritic cells, M2 macrophages, activated memory CD4 + T cells, and gamma delta T cells. In contrast, *SIGLEC10* showed negative correlations with these same immune cell types. Additionally, Tregs were inversely associated with *CYSLTR1* but positive association with *SIGLEC10*, which demonstrated concordance with the expression patterns exhibited by both target genes in the pSS set. In the IIP dataset (Fig 9H), neutrophils, monocytes, resting NK cells, and naive CD4 + T cells displayed positive correlations with *SIGLEC10* and negative correlations with *CYSLTR1*. Conversely, memory B cells, resting dendritic cells, resting mast cells, and Tregs exhibited positively associated with *CYSLTR1* and negatively correlated with *SIGLEC10*, consistent with the observed gene expression patterns. Notably, the correlation of memory B cells with both genes was consistent across both the pSS and IIP datasets, suggesting that memory B cells might be instrumental in the common mechanisms of pSS and IIP.

### 3.6. Construction of transcription factor-gene and miRNA-gene regulatory networks

**Fig 10:**
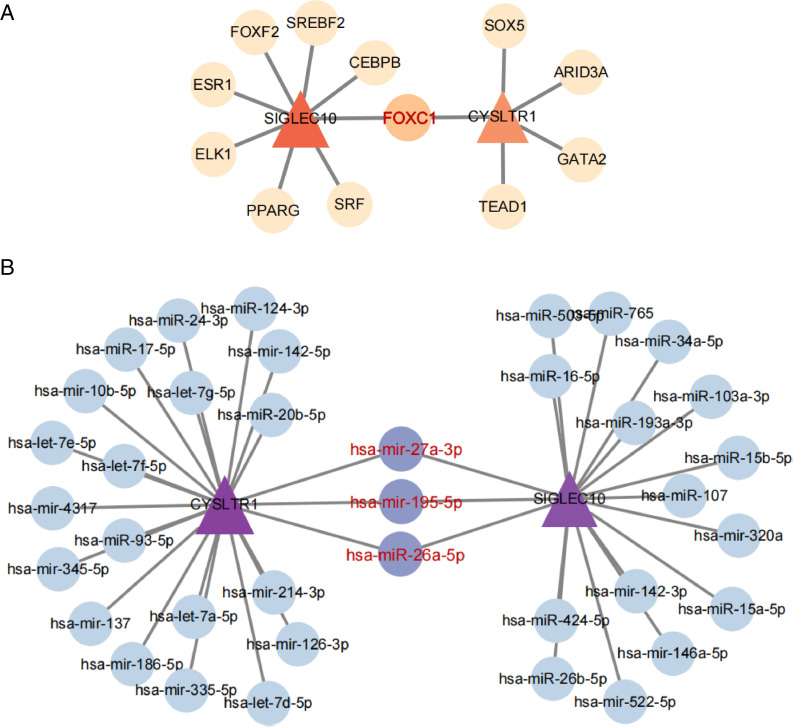
Regulatory networks involving TFs-genes and miRNAs-genes for two key genes. (A) TFs-genes regulatory network. (B) miRNAs-genes regulatory network. Key genes represented by triangles.

To gain deeper insights into disease progression, we established TFs-gene and miRNA-gene regulatory networks. The TF-gene network comprised 14 nodes and 13 edges (Fig 10A), while the gene-miRNA network included 39 nodes and 41 edges (Fig 10B). Remarkably, in the TF-gene network, FOX1 is implicated in the transcription of two key genes. In the miRNA-gene network, hsa-mir-27a-3p, hsa-mir-195-5p and hsa-miR-26a-5p interact with these genes. This suggests that these factors might jointly modulate the transcription of *CYSLTR1* and *SIGLEC10.*

### 3.7. Prediction of related drugs

We identified a total of 33 drugs associated with candidate diagnostic biomarkers in the DSigDB database. These 10 possible drug molecules which were identified through *p*-value-based ranking include Rev 5901 CTD 00002161, leukotriene D4 CTD 00007224, leukotriene C4 CTD 00007223, VERLUKAST CTD 00002469, LXB4 BOSS, montelukast CTD 00003205, zafirlukast BOSS, zafirlukast TTD 00011894, zafirlukast, zafirlukast CTD 00002560 (Table 2).

**Table 2:** The top ten related predicted drug compounds.

Term	*P*-value	Combined score	Genes
Rev 5901 CTD 00002161	0.001099	13617	CYSLTR1
leukotriene D4 CTD 00007224	0.001699	7965	CYSLTR1
leukotriene C4 CTD 00007223	0.001699	7965	CYSLTR1
VERLUKAST CTD 00002469	0.002098	6160	CYSLTR1
LXB4 BOSS	0.002298	5516	CYSLTR1
montelukast CTD 00003205	0.002398	5239	CYSLTR1
zafirlukast BOSS	0.002897	4168	CYSLTR1
zafirlukast TTD 00011894	0.0028979	4168	CYSLTR1
zafirlukast	0.0036966	3105	CYSLTR1
zafirlukast CTD 00002560	0.0042954	2590	CYSLTR1

### 3.7. Molecular docking and molecular dynamics simulation

We selected FDA-approved montelukast and zafirlukast as candidate drugs for molecular docking and molecular dynamics simulations with the core target *CYSLTR1*.

**Fig 11:**
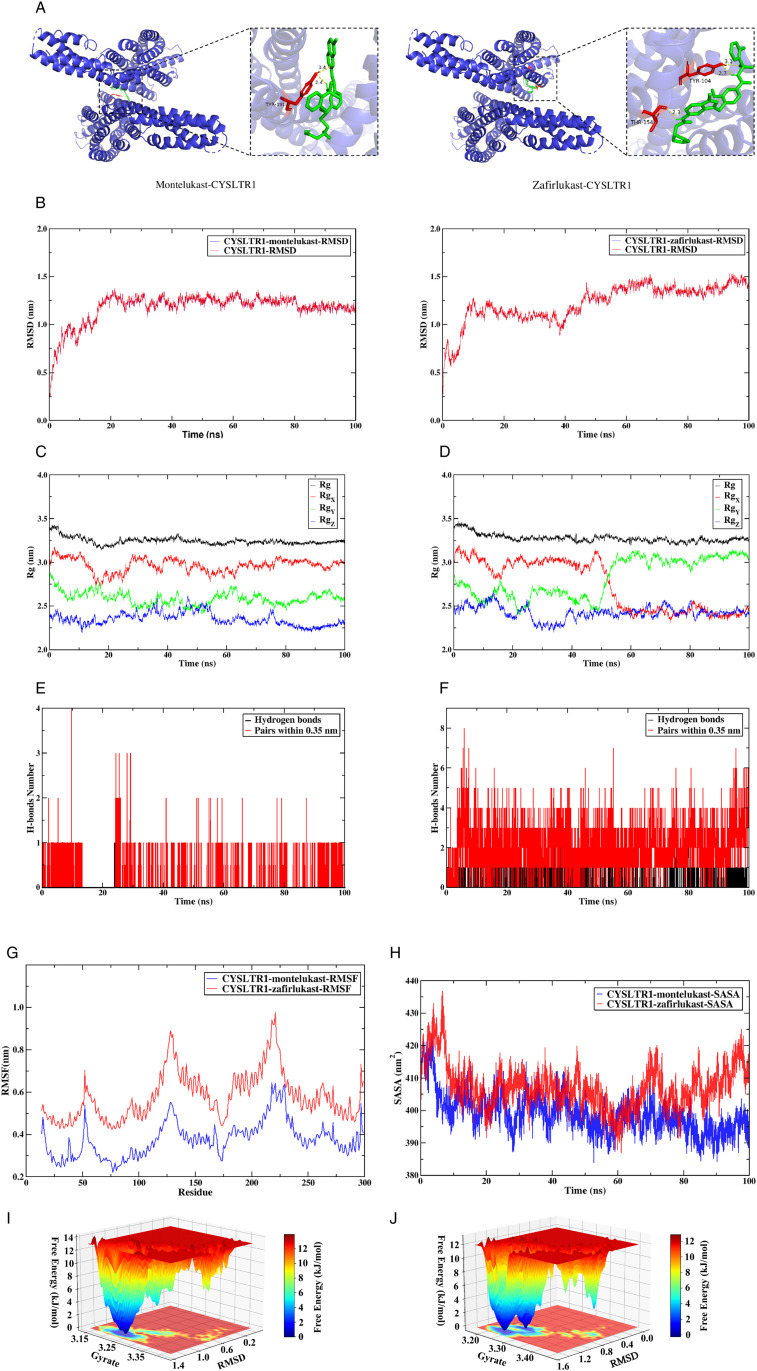
Molecular docking and molecular dynamics simulation. (A) Molecular docking poses of montelukast and zafirlukast bound to *CYSLTR1*. (B) Time-dependent RMSD changes for *CYSLTR1* complexes with montelukast and zafirlukast. (C, D) Rg profiles for montelukast-*CYSLTR1* and zafirlukast-*CYSLTR1* complexes. (E, F) Hydrogen bond formation for montelukast-*CYSLTR1* and zafirlukast-*CYSLTR1* complexes. (G) RMSF patterns of montelukast-*CYSLTR1* and zafirlukast-*CYSLTR1* complexes. (H) SASA variations montelukast-*CYSLTR1* and zafirlukast-*CYSLTR1* complexes. (I, J) Free energy landscapes of montelukast-*CYSLTR1* and zafirlukast-*CYSLTR1* conformational states.

Molecular docking results revealed binding energies of −6.78 kcal/mol for montelukast and −9.54 kcal/mol for zafirlukast with the receptor, indicating stable binding for both compounds. Visualization of the docking results provided an intuitive representation of the drug-receptor interaction patterns (Fig 11A).

Molecular dynamics simulations were employed to further validate complex stability. Results demonstrated that the *CYSLTR1*-montelukast complex rapidly reached equilibrium around 20 ns, with its RMSD values stabilizing at approximately 1.2 nm and fluctuations remaining within 0.5 nm, signifying system stability thereafter. In contrast, the *CYSLTR1*-zafirlukast complex exhibited slightly higher RMSD values and greater fluctuation amplitude, yet maintained an overall stable trend (Fig 11B). The Rg values for the montelukast complex displayed a progressive decrease, ultimately stabilizing near 3.2 nm, suggesting this ligand promotes a more compact spatial conformation of *CYSLTR1* (Fig 11C). Although the Rg values for the zafirlukast complex were marginally higher than those of montelukast, its curve also plateaued, indicating a relatively compact and stable overall conformation (Fig 11D). Hydrogen bond analysis confirmed stable hydrogen bond interactions between both ligands and the target protein (Figs 11E, F). RMSF analysis revealed significantly higher fluctuation peaks across multiple residue regions in the zafirlukast complex, whereas the montelukast complex exhibited lower overall RMSF levels with smoother fluctuations, demonstrating that montelukast binding enhances protein structural stability and restricts local flexibility (Fig 11G). SASA results showed decreased values for both complexes post-binding, indicating reduced protein surface area. Notably, the zafirlukast complex exhibited higher overall SASA values compared to montelukast, suggesting montelukast binding may induce a more compact protein structure with reduced solvent exposure (Fig 11H). The minimum free energy state for the montelukast complex clustered near RMSD = 1.2 nm and Rg = 3.25 nm (Fig 11I), with the energy well position aligning with the stable RMSD and Rg values, validating conformational reliability. The zafirlukast complex exhibited two distinct energy minima near RMSD = 1–1.3 nm and Rg = 3.20–3.30 nm (Fig 11J), separated by a low energy barrier. This suggests a potential conformational transition pathway, explaining the higher RMSD fluctuations observed in the *CYSLTR1*-zafirlukast complex.In summary, montelukast forms a structurally rigid complex with the receptor, inducing receptor compaction and exhibiting higher binding affinity. In contrast, the zafirlukast-bound receptor maintains moderate flexibility with conformational dynamics.

## 4. Discussion

Current research on pSS-ILD predominantly comprises meta-analyses and systematic reviews, with limited exploration of shared molecular pathways and genetic foundations. This study pioneers the integration of machine learning with WGCNA to elucidate common pathological mechanisms between pSS and IIP, thereby offering mechanistic insights for future pSS-ILD investigations and potential therapeutic targets to improve patient prognosis.

In this study, we identified 39 disease-associated genes that are common to both pSS and IIP. Based on GO and KEGG functional enrichment analyses, some key signaling pathways were revealed, such as chromosome segregation, oocyte maturation, and butyric acid metabolism. pSS patients have an abnormally active immune system, and the extensive proliferation of immune cells, including B cells and T cells, along with the activation and clonal expansion of lymphocytes, play essential roles in the development of pSS. Aberrant cell division could result in immune cell dysfunction and further exacerbating immune system disorders. A major feature of ILD is fibrosis of lung tissue, and proliferation of fibroblasts is a key step in the fibrotic process [30]. Signaling pathways associated with cell division, including the inactivation of the Hippo pathway, will lead to YAP/TAZ dephosphorylation in the pathological state of pulmonary fibrosis, enter the nucleus, bind to transcription factors such as TEADs, activate CTGF and CYR61, and promote the proliferation and activation of fibroblasts [31]. The incidence of pSS rises with age, and it is particularly prevalent among women in the middle-aged to elderly population, especially those between 40 and 60 years old. It is more common in [32,33], during this stage, levels of progesterone and estrogen, which are associated with oocyte maturation, decline, suggesting that abnormal hormone levels may contribute to the development of pSS, lthough this association requires further investigation. Reduced levels of butyric acid-producing intestinal bacteria are present in patients with pSS [34]. This may lead to decreased levels of SCFAs such as butyric acid, thereby affecting their role in immunomodulation. Secondly, butyric acid can maintain immune homeostasis by inhibiting dendritic cell maturation [35] and promoting Treg cell proliferation [36]. This suggests that butyrate supplementation or increasing butyric acid production by regulating the intestinal flora may have some potential therapeutic effects in alleviating the symptoms of pSS.

Then, using machine learning and Venn diagram, we obtained two candidate diagnostic markers: *CYSLTR1*, *SIGLEC10*. In comparison to the healthy control cohort, notable variations in the expression levels of these two genes were observed within the patient cohort, the expression of *CYSLTR1* was significantly elevated, whereas the expression of *SIGLEC10* was markedly reduced. Moreover, the accuracy of their diagnostic models was high according to the ROC curves, proving that the two genes have a good performance in distinguishing between diseased patients and healthy individuals.

Cysteinyl Leukotriene Receptor 1 (*CYSLTR1*), the main receptor for leukotrienes C4, D4, and E4, triggers pathological changes such as increased vascular permeability, airway smooth muscle constriction, eosinophil migration, and mucus secretion by binding to leukotrienes, playing a pivotal regulatory role in various inflammatory and fibrotic diseases. When activated by leukotrienes, this receptor initiates downstream signling pathways that stimulate pro-inflammatory cytokine release, immune cell recruitment, and tissue fibrosis progression.Recent research has revealed dual pathogenic mechanisms of *CYSLTR1* in psoriasis. Its activation promotes nuclear translocation of the NF-κB signaling pathway, elevating expression of pro-inflammatory cytokines such as TNF-α and IL-6.Through modulation of the RORγt transcription factor, it enhances Th17 cell differentiation, leading to release of key cytokines including IL-17, thereby exacerbating cutaneous inflammation and abnormal keratinocyte hyperproliferation [37]. In pulmonary fibrotic disorders, the *CYSLTR1* pathway contributes to pathogenesis via dual regulatory mechanisms. First, it facilitates the transition from inflammation to fibrosis. Activation of *CYSLTR1* on alveolar macrophages promotes TGF-β1 secretion, inducing fibroblast-to-myofibroblast differentiation and increasing extracellular matrix deposition. Second, in drug-induced lung injury models, *CYSLTR1* hyperactivation triggers mitochondrial ROS burst, resulting in apoptosis of hepatic sinusoidal endothelial cells-a process directly linked to upregulated ALOX5/5-LOX pathway activity [38]. Based on these findings, we propose that *CYSLTR1* likely serves as a pivotal hub in the pathogenesis of pSS-ILD, driving disease progression by mediating the transition from inflammatory responses to fibrotic phenotypes. This mechanistic insight suggests that *CYSLTR1* functions not only as a core molecular switch connecting early inflammation with advanced fibrosis, but also represents a novel therapeutic target for intervening in disease outcomes. Consequently, further experimental investigations are warranted to validate these observations.

Sialic acid-binding Ig-like lectin 10 (*SIGLEC10*), a member of the Siglec family of immunoreceptors, is often thought to interact with CD24, triggering an inhibitory signaling cascade that suppresses the destructive inflammatory response in sepsis, infections, hepatic injuries, and chronic graft-versus-host reactions [39]. We propose that during the progression of pSS-ILD, downregulated *SIGLEC10* expression may enhance autoimmune responses by releasing immunosuppression. *SIGLEC10* is also critically involved in tumor immune evasion [40]. Multiple studies have demonstrated that *SIGLEC10* serves as a significant immunosuppressive mediator in gastric, hepatocellular and cervical cancers, and helps tumors evade immune surveillance by interfering with the normal function of NK cells and inhibiting cross-presentation of dendritic cells [41–43]. Thus, in autoimmune diseases, functional impairment of *SIGLEC10* may similarly disrupt immune tolerance to self-antigens, leading to amplified aggressive immune responses. These findings indicate that as an inhibitory receptor on multiple immune cells, *SIGLEC10* potentially contributes to pSS-ILD progression through its downregulation-induced immune hyperactivation.

In the immune microenvironment of pSS and IIP, elevated proportions of memory B cells and activated memory CD4 + T cells were observed, whereas a marked reduction in resting NK cells was evident. In several studies, memory B cells and activated memory CD4 + T cells exhibited a marked augmentation frequency in patients with pSS relative to healthy controls [44,45]. Memory B cells possess the capacity to swiftly transition into plasma cells, subsequently generating a substantial quantity of autoantibodies, such as anti-SSA and anti-SSB antibodies [46], when they encounter antigen again, which are intricately linked to the pathogenesis of pSS. In the course of the disease progression of pSS, aerobic glycolysis of activated memory CD4 + T cells is enhanced, leading to the overproduction of IFN-γ and IL-17A, which promotes the expansion and maturation processes of B lymphocytes and exacerbates the autoimmune response [47]. In ILD disease, these two types of immune cells interact with alveolar epithelial cells and fibroblasts, exacerbating the inflammatory response and fibrosis of lung tissues [48,49]. The functional and mechanistic roles of NK cells in pSS and ILD have yet to be fully elucidated across diverse pathophysiological contexts. Considering their capacity to directly lyse target cells and secrete cytokines, NK cells may contribute to the perpetuation and amplification of autoimmune responses [50]. In summary, these immune cells may be involved in the transition from immune activation to fibrosis during the course of pSS-ILD.

Our results suggest that FOXC1, hsa-mir-27a-3p, hsa-mir-195-5p, and hsa-miR-26a-5p may be important regulators of the two shared genes. FOXC1 demonstrates elevated expression across a wide spectrum of cancers and is intricately associated with tumor invasion, metastasis, and clinical outcomes. For example, in non-small cell lung cancer(NSCLC), FOXC1 drives tumor advancement and suppresses the immune microenvironment through its mediation of LINC00301 in regulating the HIF1α pathway [51]. It has been discovered that Hsa-mir-27a-3p exerts a significant regulatory function during the course of colorectal cancer development. For example, Hsa-mir-27a-3p might decrease the synthesis of collagen I and III in lung fibroblasts through suppressing the Wnt3a/β-catenin pathway in pulmonary fibrosis [52], which may be relevant to the disease progression of ILD that we studied. In addition, by regulating the RXRα/β-catenin pathway, hsa-mir-27a-3p is capable of influencing various vital activities of cancer cells, such as proliferation, migration and invasion. Studies have shown that hsa-mir-195-5p can inhibit the autophagy process of lung adenocarcinoma cells and reduce their resistance to gemcitabine by targeting the E2F7/CEP55 signaling axis [53]. Hsa-mir-195-5pa serves as a biological indicator for assessing the risk of lung cancer, and also regulate the expression of Indian hedgehog to control the process of osteogenic differentiation in human adipose-derived mesenchymal stem cells [54]. Hsa-miR-26a-5p influences the osteogenic differentiation of chondrocytes in arthritis through the activation of the NF-κB signaling pathway [55]. In colon cancer (CRC), the expression level of hsa-miR-26a-5p is reduced, and it regulates the expression of MTDH by interacting with SNHG5, a long chain non-coding RNA, thus affecting the proliferative and metastasis of CRC cells [56]. In summary, hsa-mir-27a-3p may have an effect on pulmonary fibrosis in pSS-ILD disease progression, and FOXC1, hsa-mir-195-5p and hsa-miR-26a-5p have not been sufficiently investigated in pSS and ILD, which deserves further study.

Our drug prediction analysis identified montelukast as a promising therapeutic candidate for pSS-ILD. Molecular docking and dynamics simulations revealed a compact, rigid complex formation between montelukast and the target receptor, indicative of potent inhibitory effects potentially halting disease progression. Given the pivotal role of *CYSLTR1* in autoimmune pathogenesis, exploration of CYSLTR1 antagonists for therapeutic repurposing has gained significant momentum.One study demonstrated, *CYSLTR1* expression is increased in psoriatic skin lesions, and studies have shown that montelukast, a *CYSLTR1* antagonist, has demonstrated significant potential in the therapeutic management of diverse connective tissue disorders. For example, it can address psoriasis by suppressing the differentiation of Th17 cells [37]. In rheumatoid arthritis, *CYSLTR1* expression was also found to be up-regulated [57]. Under the influence of montelukast, the secretion of metalloproteinases, such as MMP-3, MMP-13, IL-8 and IL-6, by synovial fibroblasts is diminished, thereby attenuating joint inflammation [58]. Furthermore, in multiple sclerosis, montelukast might treat the disease by impeding the migration of Th17 cells [59]. In contrast, zafirlukast—currently indicated for asthma management—represents an unexplored therapeutic candidate for CTD-ILD. Our simulations demonstrate its formation of a moderately flexible receptor complex with relaxed structural constraints, suggesting tissue-selective disease suppression through allosteric blockade. *CYSLTR1* antagonists demonstrate significant therapeutic potential across autoimmune disorders, with montelukast emerging as a particularly viable strategy for pSS-ILD pending further in vitro/in vivo validation and optimization.

Additionally, our study is subject to some limitations. First, constrained by the limited sample size of the datasets and the absence of data from patients with concurrent pSS and ILD, the statistical power and generalizability of our findings may be limited. Future studies could expand sample sizes and systematically incorporate comorbidity data to enhance the reliability and clinical applicability of conclusions. Second, due to missing clinical variables in public databases, we were unable to adjust for covariates such as age and sex, potentially introducing confounding effects. We recommend validating findings in cohorts with complete clinical records to address this limitation. Third, due to the limited availability of high-throughput data meeting the inclusion criteria in online databases and to avoid the impact of inter-platform batch effects and differences in gene coverage on the reliability of conclusions, this study exclusively incorporated microarray datasets. Subsequent research is recommended to validate the findings using bulk RNA-seq datasets, which offer greater sensitivity, dynamic range, and quantification accuracy. Additionally, experimental validation through in vitro and in vivo models was beyond the scope of this study. The biological relevance of our findings would benefit from further exploration in subsequent laboratory studies and clinical practice. Nevertheless, this work identifies potential biomarkers for pSS-ILD diagnosis. Delving into shared molecular mechanisms between these conditions will not only elucidate the nature of disease comorbidity but also provide a critical theoretical foundation for developing cross-disease precision diagnostic tools and targeted therapies, ultimately improving patient prognosis.
